# Evaluation of the efficacy of exogenous enzymes on nutrient digestibility of ducks *in vitro* and *in vivo*

**DOI:** 10.3389/fvets.2026.1939685

**Published:** 2026-09-09

**Authors:** Hai-lin Su, Qi Hu, Shi-cai Qu, Jie Jin, Hua-qi Zhang, Chao-yong Wang, Wen-ce Wang, Songbai Liu, Yong-wen Zhu

**Affiliations:** 1 State Key Laboratory of Livestock and Poultry Breeding, Guangdong Provincial Key Laboratory of Animal Nutrition and Regulation, College of Animal Science, South China Agricultural University, Guangzhou, China; 2 Guangdong Laboratory for Modern Agricultural Science and Technology Innovation Center, Jinji Farm Ecological Agriculture Development Co., Ltd., Anshun, China; 3 Key Open Laboratory of Chinese Veterinary Medicine of State Ethnic Affairs Commission, Tongren Polytechnic University, Tongren, China; 4 Wens Food Group Co. Ltd., Yunfu, China

**Keywords:** duck, energy digestibility, exogenous enzyme, *in vitro*, *in vivo*

## Abstract

Our study aimed to investigate the efficacy of exogenous enzymes on the nutrient digestibility of ducks *in vitro* and *in vivo*. First, a two-stage simulated digestion assay for ducks was used to evaluate the *in vitro* digestibility of dry matter (IVDMD) and energy (IVED) of wheat, rice, and broken rice samples, as well as cottonseed meal (CSM), rapeseed meal (RSM), and peanut meal (PNM) samples. Then, the improvements in IVDMD and IVED in wheat 3, rice 4, CSM 3, and RSM 3—the ingredients with the lowest nutritive values—with and without exogenous enzyme supplementation were evaluated. To verify the efficacy of exogenous enzymes on nutrient digestibility *in vivo*, four diets, including a positive control (PC) diet (corn–wheat–soybean meal–RSM-based diet), a negative control (NC) diet with a low nutrient level, an NC diet + non-starch polysaccharide (NSP) enzymes, and an NC diet + NSP enzymes + protease, were fed to Cherry Valley ducks from days 14 to days 42. Compared with the control, single-NSP-enzyme supplementation increased IVDMD by 3.27% and IVED by 2.85%, and the combined-enzyme addition increased IVDMD by 6.04% and IVED by 6.20% in wheat 3, respectively (*p* < 0.05). Supplementation of NSP enzymes alone or with the protease increased the final BW and average daily gain (ADG) and improved the feed/gain ratio (F/G), as well as the digestibility of DM, energy, crude protein, and some amino acids (Thr, Met, Ile, Lys, Arg) in ducks fed the NC diet (*p* < 0.01). In conclusion, exogenous enzymes improved the nutrient digestibility of wheat and RSM as well as the corn–wheat–soybean meal–RSM basal diet *in vitro* and *in vivo*, respectively, contributing to the improvement in growth performance of ducks fed a diet with reduced nutrient levels. The consistency of the beneficial effects of exogenous enzymes between *in vitro* and *in vivo* conditions suggests that an *in vitro* assay could be developed into a quick and reliable method for assessing the efficacy of exogenous enzymes in ducks.

## 1 Introduction

Due to the unreliable supply and increasing cost of conventional feedstuffs, wheat, rapeseed meal (RSM), and cottonseed meal (CSM) have been used to partially substitute corn and soybean meal in poultry diets (1). However, the main limitation in the application of these feedstuffs is the presence of a wide range of anti-nutritional factors, such as glucosinolates in RSM, non-starch polysaccharides (NSP) in wheat, and gossypol in CSM (2, 3), leading to decreased organic matter and energy digestibility (4, 5). Exogenous enzymes have been widely used as feed additives to degrade the bonds between antinutritional factors and nutrients in feedstuffs. The use of enzymes that target specific anti-nutrient factors in certain feed ingredients can release more nutrients from the diet to be digested and absorbed by animals. For example, the soluble NSPs in wheat bran can be effectively degraded by xylanase and β-glucanase to release more available starch and protein (6). In addition, there is an additive beneficial effect on growth performance and nutrient digestibility in corn- and wheat-based diets from the supplementation of protease on top of xylanase and amylase (7). Therefore, the wide variety of commercially available feed enzymes makes it critically important to accurately assess the efficacy of exogenous enzymes, which depends on the animal species, substrate specificity, activity, stability, and other factors (8). A two-stage *in vitro* digestion assay has been shown to evaluate the efficacy of exogenous enzymes with acceptable accuracy (9, 10), displaying an alternative method to improve our knowledge of enzyme action along the digestive tract. Previous studies on the evaluation of β-glucanase and xylanase using *in vitro* methods have shown results similar to those obtained *in vivo* for the increased available ME of wheat (11, 12). However, the response and correlation of enzyme addition in feedstuffs between *in vitro* and *in vivo* conditions are not yet fully understood due to the variability in ingredient types and characteristics, such as their NSP content, structure, and the substrate specificity of exogenous enzymes. Given the inconsistent efficacy of exogenous NSP enzymes used alone or combined with protease for different plant feed ingredients in duck diets, the objective of the present study was to adopt a two-stage *in vitro* simulated digestion assay to evaluate the efficacy of exogenous NSP enzymes, either individually or in combination with protease, on the energy digestibility of wheat, rice, broken rice, CSM, RSM, and peanut meal (PNM). Furthermore, *in vivo* feeding experiments were performed to verify the beneficial effects of exogenous enzyme supplementation on growth performance, slaughter performance, and nutrient digestibility of Cherry Valley ducks from days 14 to 42, and to test the hypothesis combined supplementation of exogenous NSP enzymes and protease presents greater improvements in nutrient utilization than single-enzyme treatments for meat ducks.

## 2 Materials and methods

### 2.1 Nutrient composition

Twenty-one samples of six different unconventional feedstuffs, including three energy sources of cereals (cereal grains: wheat, rice, and broken rice) and three protein meals (CSM, RSM, and PNM), were evaluated for the digestibility of DM and gross energy (GE). Four samples of each energy ingredient and three samples of each protein ingredient were sourced from different provinces in China. Wheat samples 1–4 were collected from the provinces of Jiangsu (medium crude fiber, CF, 1.7–1.9%), Anhui (very low CF, <1.5%), Liaoning (high CF, >1.9%), and Shanxi (low CF, 1.5–1.7%) in China, respectively. Rice samples 1–4 were collected from Zhejiang (very low CF, <8%), Guangdong (medium CF, 9–10%), Sichuan (low CF, 8–9%), and Jiangxi (high CF, >10%) in China, respectively. Broken rice samples 1–4 were collected from the provinces of Liaoning (low CF, < 1%), Guangdong (medium CF, 1–1.5%), Sichuan (medium CF, 1–1.5%), and Jiangxi (high CF, >1.5%) in China, respectively. CSM samples 1–3 were collected from the Sichuan (medium gossypol), Hebei (low gossypol), and Jiangsu (high gossypol) provinces, respectively. RSM samples 1–3 were collected from the provinces of Jiangsu (double zero RSM, with low levels of glucosinolates and erucic acid), Shandong (single zero normal RSM), and Anhui (normal RSM) in China, respectively. PNM samples 1–3 were collected from the provinces of Gansu, Shandong, and Hebei in China, respectively. All samples were ground to pass through a 1-mm screen in a Wiley mill (Model 4; Thomas Scientific, Swedesboro, NJ, United States). Each sample was analyzed in triplicate for the contents of GE, CP, ash, CF, neutral detergent fiber (NDF), acid detergent fiber (ADF), calcium (Ca), and total phosphorus (TP) on a DM basis.

### 2.2 *In vitro* assays

In Exp. 1, a computer-controlled simulated digestion system was designed to automatically process the *in vitro* gastrointestinal digestion procedure for ducks. A total of 21 distinct feedstuff sample units originating from different provinces were analyzed in the present study. The nutrient digestibility was determined with five replicates of each sample of wheat, rice, broken rice, CSM, RSM, and PNM ingredients according to the procedure described in detail by Zhao et al. (10). Briefly, 2 g of the energy cereal samples or 1 g of the protein meal samples were incubated with a gastric buffer solution containing 1,550 U/mL pepsin at pH 3.5 (Sigma 10,070, Sigma-Aldrich Co., St. Louis, MO) for 4 h in dialysis tubing (Membra-cel MD 44–14, 14,000 Dalton, Viskase Companies Inc., Darien, IL) at 42 °C with occasional vortexing to simulate the peptic or gastric phase. After gastric digestion, small intestinal digestion was initiated with the upper small intestinal buffer solution circularly pumped into digestion chambers for 0.5 h; then, 2 mL of simulated small intestinal fluid containing 401.56 U/mL amylase (Sigma A3306, Sigma-Aldrich Co., St. Louis, MO), 108.75 U/mL trypsin (Amersco 0785, Amersco Inc., Solon, OH), and 39.01 U/mL chymotrypsin (Amersco 0164, Amersco Inc., Solon, OH) at pH 7.0 was injected into each digestion chamber and the buffer solution continued to circulate for 7.5 h at 42 °C. When the upper small intestinal digestion was finished, the lower small intestinal buffer solution was pumped into the digestion chambers for 7.5 h at 42 °C. The small intestinal buffer solutions were prepared with 800,000 U/L penicillin to prevent microbial degradation. After the simulated digestion was completed, the undigested residues from each replicate were defatted and dried at 105 °C for 5 h to a constant weight for the measurement of nutrient digestibility. Then, the samples of wheat 3, rice 4, CSM 3, and RSM 3, which exhibited the lowest nutrient digestibility among the wheat, rice, CSM, and RSM samples, were screened for the evaluation of the improvements in DM and energy digestibility conferred by enzyme addition. A single-factor experiment was designed to study the effects of single and combined enzymes on the improvement of nutrient digestibility in feedstuffs. Each sample of wheat, rice, CSM, and RSM ingredients with three enzyme preparations (no enzymes as the control, control + NSP enzymes, and control + NSP enzymes + protease) was subjected to the computer-controlled, two-stage *in vitro* digestion assay. The DM and energy digestibility were determined with five replicates of each treatment. All enzyme products used in the present study were acquired from commercial suppliers specializing in nutritional supplements. The NSP enzyme activities were assayed via colorimetric determination of reducing sugars to provide 1,500 U xylanase/kg of diet and 2,500 U β-glucanase/kg of diet, while protease activity was assayed via colorimetric determination of tyrosine equivalents released from casein to provide 5,000 U protease/kg of diet. As described previously, the sample residues were collected after the gastric and small intestinal *in vitro* digestions, and were defatted and dried for the measurement of DM and energy digestibility.

### 2.3 *In vivo* assays

In Exp. 2, a total of 700 1-day-old male Cherry Valley ducks (Guangdong Academy of Agricultural Sciences, Guangzhou, China) were fed a commercial diet (2,900 kcal/kg of ME, 20.05% CP, 1.00% Ca, 0.45% non-phytate P, 1.14% Lys, 0.50% Met) to 14 days of age. On day 14, a total of 600 healthy ducks were selected and divided into four treatment groups based on similar BW, each with six replicates of 25 ducks per replicate. The experimental period was from 14 to 42 days of age. The four experimental diets included a basal diet without any exogenous enzymes enzyme additions as a PC diet (corn–wheat–soybean meal–RSM basal diet) with adequate nutrient levels (3,002 kcal/kg ME, 18.00% CP, 0.86% Ca, 0.31% non-phytate P, 0.94% Lys, 0.47% Met) and a NC diet with a lower nutritional levels (2,751 kcal/kg ME, 16.06% CP, 0.88% Ca, 0.34% non-phytate P, 0.79% Lys, 0.37% Met), as well as the NC diet supplemented with NSP enzymes (NC + NSP enzymes) and the NC diet supplemented with NSP enzymes and protease (NC + NSP enzymes + protease). Wheat 3 and RSM 3 were used to partially substitute corn and soybean meal in the basal diet to meet the nutritional requirements recommended by the National Agriculture Industry Standard in China (13) for meat-type ducks aged 14 to 42 days. The ingredient composition and nutritional levels of the experimental diets are presented in Table 1. As described in Exp.1, NSP enzymes (1,500 U/kg xylanase and 2,500 U/kg β-glucanase) and protease (5,000 U/kg) were added to the feed additive premix to formulate the complete diet. Before the initiation of the experiment, the CP, Ca, and TP contents in the experimental diet samples were analytically determined to confirm the calculated values in the diets. Birds were allowed ad libitum access to diets and tap water during the experimental period. Daylight was eliminated and replaced with an 18-h light: 6-h dark photoperiod from incandescent bulbs. The room temperature was kept at 33 °C from days 1 to 3, and then it was reduced gradually to approximately 25 °C by 14 day of age and was maintained in an environmentally controlled room (25 °C) during 14–42 days of age. At day 42, after a 12-h feed withdrawal, birds were weighed, and feed consumption and mortality were recorded for each replicate pen. The final BW, average daily gain (ADG), average daily feed intake (ADFI), feed/gain ratio (F/G), and daily mortality were calculated. Based on the average BW of birds in each replicate pen, two birds from each pen were individually weighed and then euthanized via inhalation of 20% CO₂, which was introduced at a displacement rate of 30% of the euthanasia chamber volume per minute. Once the birds became motionless, they were observed for at least 3 min and immediately bled. After the heads and feet were removed, the carcasses were eviscerated and weighed to determine the dressing percentage. Abdominal fat, the left breast, and leg muscles were removed and weighed to determine the percentages of abdominal fat, breast muscle, and leg muscle, respectively. On days 43 and 44, all experimental diets were mixed with titanium dioxide (0.4%) as an indigestible marker and were fed as a mash to the rest of the birds in each replicate cage. The excreta samples were collected from each pen over 3 h/day. After each collection, fresh excreta subsamples were immediately transferred into sealed plastic bags and preserved at −20 °C for frozen storage. Upon completion of the entire collection procedure, all frozen subsamples from each pen were fully thawed, thoroughly pooled and mixed, and the composite sample was then placed in an oven for air-drying at 60 °C for 4 days.

**Table 1:** Feed ingredient and nutrient composition of experimental diets (%, as-fed basis).

Items, %	d 1–14	d 14–42	
		PC	NC
Ingredient			
Corn	45.50	44.30	49.00
Wheat	15.00	20.00	20.00
Soybean meal	29.00	19.10	11.50
Rapeseed meal	4.00	8.00	8.00
Wheat bran	0.35	1.53	7.94
Soybean oil	2.00	3.50	0.00
Calcium hydrogen phosphate	1.30	0.68	0.70
Stone powder	1.30	1.30	1.30
Premix^1^	1.00	1.00	1.00
Lys	0.20	0.20	0.25
Met	0.19	0.19	0.11
Salt	0.16	0.20	0.20
Nutritional level^2,3^			
Metabolizable energy, kcal/kg	2,900	3,002	2,751
Crude protein	20.05	18.0	16.0
Crude fiber	3.50	3.31	3.63
Crude fat	4.50	5.86	2.70
Starch	38.41	40.55	44.72
Calcium	1.00	0.86	0.88
Total phosphorus	0.70	0.57	0.55
Available phosphorus	0.45	0.31	0.34
Digestible Lys	1.14	0.94	0.79
Digestible Met	0.50	0.47	0.37
Digestible Tryptophan	0.27	0.21	0.18
Digestible Histidine	0.56	0.45	0.39
Digestible Arginine	1.00	1.10	0.92
Digestible Leucine	1.33	1.41	1.25
Digestible Isoleucine	0.75	0.69	0.58
Digestible Phenylalanine	1.06	0.84	0.73
Digestible Valine	0.86	0.83	0.73
Digestible Threonine	0.82	0.65	0.56

^1^Provided per kilogram of complete diet during 1–14 d of age: vitamin A, 13000 IU; vitamin D_3_, 3,000 IU; vitamin K_3_, 4 mg; vitamin E, 20 IU; vitamin B_1_, 5 mg; vitamin B_2_, 15 mg; vitamin B_6_, 8 mg; vitamin B_12_, 0.05 mg; calcium-D-pantothenate, 15 mg; niacin, 50 mg; folate, 1.5 mg; biotin, 0.3 mg; choline (choline chloride), 1,000 mg; Fe (FeSO_4_·7H_2_O), 60 mg; Cu (CuSO_4_·5H_2_O), 8 mg; Mn (MnSO_4_·H_2_O), 80 mg; Zn (ZnSO_4_·7H_2_O), 70 mg; Se (NaSeO_3_), 0.3 mg; I (KI), 0.4 mg.

^1^Provided per kilogram of complete diet during 14–42 d of age: vitamin A, 10000 IU; vitamin D_3_, 3,000 IU; vitamin K_3_, 3 mg; vitamin E, 15 IU; vitamin B_1_, 3 mg; vitamin B_2_, 13 mg; vitamin B_6_, 7 mg; vitamin B_12_, 0.03 mg; calcium-D-pantothenate, 15 mg; niacin, 45 mg; folate, 1.5 mg; biotin, 0.2 mg; choline (choline chloride), 700 mg; Fe (FeSO_4_·7H_2_O), 40 mg; Cu (CuSO_4_·5H_2_O), 8 mg; Mn (MnSO_4_·H_2_O), 60 mg; Zn (ZnSO_4_·7H_2_O), 60 mg; Se (NaSeO_3_), 0.3 mg; I (KI), 0.3 mg.

^2^Calculated values.

^3^The content of xylanase, β-glucanase, and protease in the basal diet was below the detectable level.

### 2.4 Sample preparation and analysis

The dried excreta samples and diet samples were ground finely in a centrifugal mill (model ZM200; Retsch GmbH, Haan, Germany) to pass through a 0.75-mm screen. DM was measured according to the Association of Official Analytical Chemists standard procedures (AOAC Method 930.15; AOAC, 2007) at 135 °C for 2 h. The GE values were measured using an isoperibol oxygen bomb calorimeter (Kalorimeter C 7000 Prozesso, IKA-Weke, Staufen, Germany). The determination of CP content was performed with the Kjeldahl method (Method 984.13; AOAC, 2007) on a Kjeltec™ 8,400 analyzer (FOSS Inc., Eden Prairie, MN). The ash content was analyzed using a muffle furnace at 600 °C for 2 h (Method 942.05; AOAC, 1990). The contents of Ca and P were analyzed by inductively coupled plasma optical emission spectroscopy (Method 985.01; AOAC, 2007). The contents of NDF (without sodium sulfite and α -amylase, expressed inclusive of residual ash) and ADF (expressed inclusive of residual ash) were measured as described by Van Soest et al. (14). Amino acids (AAs) in the diets and excreta samples were analyzed using an AA analyzer (model L-8500A; Hitachi Ltd., Chyoudaku, Tokyo, Japan).

### 2.5 Calculation and statistical analysis

*In vitro* digestibility of dry matter (IVDMD) and energy (IVED) were calculated using the following equations as described in a previous study (15):

IVDMD = (sample DM weight – defatted residue DM weight)/sample DM weight; IVED = [(sample DM weight × sample GE) – (defatted residue DM weight × defatted residue GE)/(sample DM weight × sample GE)].

The apparent total tract digestibility of DM, GE, CP, and AAs in the diets was calculated according to the following formula as described in a previous study (15):

Nutrient digestibility (%) = {1 – ((TiO_2_ feed/TiO_2_ excreta) × (Nutrient excreta/Nutrient feed))} × 100.

The data were subjected to a one-way ANOVA using the General Linear Model procedure of SAS 9.2 (43). Each replicate served as the experimental unit for all statistical analyses. Treatment comparisons for significant differences were tested by the LSD method. Significant differences were set at *p* ≤ 0.05.

## 3 Results

### 3.1 Digestibility of dry matter and energy of feedstuffs

The *in vitro* digestibility of dry matter and energy of wheat, rice, broken rice, CSM, RSM, and PNM samples from different provinces were measured using an *in vitro* computer-controlled simulated digestion system (Table 2). When comparing samples of the same feedstuff collected across different provinces, the IVDMD and IVED values in wheat 3, rice 4, and broken rice 4 were significantly lower than those of the other corresponding samples (*p* < 0.05), while the IVDMD and IVED values in CSM 3, RSM 3, and PNM 3 were likewise significantly lower than those of the other samples within their respective feedstuff categories (*p* < 0.05).

**Table 2:** *In vitro* digestibility of dry matter and energy of wheat, rice, broken rice, cottonseed meal, rapeseed meal, and peanut meal samples from different locations^1^.

Items^2^	1	2	3	4	SEM	*p*-value
Wheat						
IVDMD^3^	80.3^a^	78.8^a^	75.6^b^	81.4^a^	0.64	<0.01
IVED^3^	82.4^a^	81.9^a^	79.2^b^	83.0^a^	0.83	0.05
Rice						
IVDMD^3^	72.5^a^	76.4^b^	70.1^c^	66.6^d^	0.34	<0.01
IVED^3^	74.3^b^	78.1^a^	71.6^b^	65.8^c^	0.57	<0.01
Broken rice						
IVDMD^3^	87.4^a^	80.5^a^	83.7^b^	76.9^b^	0.94	<0.01
IVED^3^	89.5^a^	82.8^a^	85.6^b^	78.3^c^	0.83	<0.01
CSM						
IVDMD^3^	58.5^ab^	62.4^a^	55.4^b^		0.44	<0.01
IVED^3^	58.7^a^	62.2^b^	55.2^b^		0.34	<0.01
RSM						
IVDMD^3^	61.6^b^	66.8^a^	54.0^c^		0.28	<0.01
IVED^3^	62.1^b^	67.7^a^	57.3^c^		0.30	<0.01
PNM						
IVDMD^3^	72.7^a^	72.2^a^	68.8^b^		0.60	0.02
IVED^3^	79.4^a^	79.2^a^	75.1^b^		0.68	0.02

^a–c^Means within a row lacking a common superscript letter are different (*p* < 0.05).

^1^Wheat samples 1–4 were collected from the Jiangsu, Anhui, Liaoning, and Shanxi provinces of China, respectively.

Rice samples 1–4 were collected from Zhejiang, Guangdong, Sichuan, and Jiangxi provinces of China, respectively.

Broken rice samples 1–4 were collected from Liaoning, Guangdong, Sichuan, and Jiangxi provinces of China, respectively; CSM samples 1–3 were collected from Sichuan, Hebei, and Jiangsu provinces of China, respectively; RSM samples 1–3 were collected from Jiangsu, Shandong, and Anhui provinces of China, respectively; PNM samples 1–3 were collected from Gansu, Shandong, and Hebei provinces of China, respectively.

^2^The values represent mean of five replicates per sample.

^3^IVDMD = (sample dry matter weight − defatted residue dry matter weight)/sample dry matte weight; IVED = [(sample dry matter weight × sample gross energy) − (defatted residue dry matter weight × defatted residue gross energy)]/(sample dry matter weight × sample gross energy).

IVDMD, *in vitro* dry matter digestibility; IVED, *in vitro* energy digestibility; IVDE, *in vitro* digestible energy; SEM, standard error of means.

### 3.2 Effect of enzyme addition on nutrient digestibility *in vitro*

The *in vitro* digestibility of dry matter and energy of wheat 3, rice 4, CSM 3, and RSM 3 samples—exhibiting the lowest IVED values—was measured following exogenous enzyme addition (Table 3). Compared with the control, single-NSP-enzyme supplementation increased IVDMD by 3.27% and IVED by 2.85%, and the combined-enzyme addition increased IVDMD by 6.04% and IVED by 6.20% in wheat 3, respectively. However, no significant effects of NSP-enzyme supplementation on IVDMD and IVED were observed in the screened rice 4, CSM 3, and RSM 3 samples (*p* > 0.05).

**Table 3:** *In vitro* digestibility of dry matter and energy of wheat, rice, cottonseed meal, and rapeseed meal samples with low *in vitro* energy digestibility value by enzyme addition^1^.

Sample	Items^2^	Control	NSP enzyme	NSP enzyme + protease	SEM	*P*-value
Wheat 3	IVDMD^3^	79.4^c^	82.0^b^	84.2^a^	0.15	0.005
	IVED^3^	80.6^c^	82.9^b^	85.6^a^	0.66	0.002
Rice 4	IVDMD^3^	63.9	64.9	65.7	0.10	0.06
	IVED^3^	65.4	66.4	67.6	0.60	0.07
CSM 3	IVDMD^3^	47.2	48.9	49.6	0.80	0.14
	IVED^3^	51.3	51.3	51.4	0.60	0.29
RSM 3	IVDMD^3^	55.5	55.8	58.2	0.73	0.052
	IVED^3^	62.2	62.8	64.1	0.75	0.22

^a–c^Means within a row lacking a common superscript letter are different (*p* < 0.05).

^1^Wheat 3, Rice 4, CSM 3, and RSM 3 were collected from Liaoning, Jiangxi, Jiangsu, and Anhui provinces of China, respectively.

^2^The values represent mean of five replicates per sample.

The NSP enzymes were provided with 1,500 U xylanase/kg diet and 2,500 U glucanase/kg diet. Protease was provided with 5,000 U/kg of diet.

^3^IVDMD = (sample dry matter weight − defatted residue dry matter weight)/sample dry matte weight; IVED = [(sample dry matter weight × sample gross energy) − (defatted residue dry matter weight × defatted residue gross energy)]/(sample dry matter weight × sample gross energy). IVDMD, *in vitro* dry matter digestibility; IVED, *in vitro* energy digestibility; SEM, standard error of means; NSP, NSP enzymes addition.

### 3.3 Effect of enzyme addition on growth performance and slaughter performance *in vivo*

Ducks fed the PC diet had greater (*p* < 0.01) final BW, ADG, ADFI, and abdominal fat percentages, as well as a lower F/G, than ducks fed the NC diet regardless of enzyme supplementation from 14 to 42 days of age (Tables 4, 5). The addition of exogenous NSP enzymes alone and in combination with protease increased the final BW of ducks fed the NC diet by 3.98 and 4.21%, respectively, and increased their ADG by 4.48 and 5.34%, respectively (*p* < 0.01). Supplementing NSP enzymes in combination with protease reduced F/G by 7.25% (*p* < 0.01). However, no significant exogenous enzyme effects were found in ADFI from 14 to 42 days of age, or on the slaughter performance and abdominal fat percentages of 42-d-old ducks among the NC groups (*p* > 0.05, Table 5).

**Table 4:** Effects of enzyme addition on growth performance of Cherry Valley ducks at d 14–42^1^.

Items^2^	PC	NC	NC + NSP enzyme	NC + NSP enzyme + protease	SEM	*P*-value
Final BW, g	2694^a^	2515^c^	2615^b^	2621^b^	25.05	<0.01
ADG, g/d/bird	80.27^a^	72.62^c^	75.87^b^	76.50^b^	0.70	<0.01
ADFI, g/d/bird	182.5^b^	200.5^a^	201.4^a^	195.8^ab^	4.38	0.01
F/G	2.27^c^	2.76^a^	2.65^ab^	2.56^b^	0.05	<0.01

^1^Each value represents the mean of 6 replicates.

The NSP enzymes were provided with 1,500 U xylanase/kg diet and 2,500 U glucanase/kg diet. Protease was provided with 5,000 U/kg of diet.

^a–c^Means with different superscripts within the same column differ significantly (*p* < 0.05).

^2^PC, positive control; NC, negative control; NSP, NSP enzymes addition; SEM, standard error of mean; BW, body weight; ADG, average daily gain; ADFI, average daily feed intake; F/G, feed/gain ratio.

**Table 5:** Effects of enzyme addition on slaughter performance of Cherry Valley ducks at d 42^1^.

Items^2^, %	PC	NC	NC + NSP enzyme	NC + NSP enzyme + protease	SEM	*P*-value
Dressing percentage	85.13	84.55	85.76	84.91	0.92	0.82
Half-eviscerated carcass yield	82.74	82.82	83.98	83.00	1.08	0.84
Eviscerated carcass yield	72.81	75.08	75.10	74.49	1.40	0.63
Breast yield	12.57	10.86	11.57	10.79	0.72	0.30
Thigh yield	11.80	11.37	12.80	11.44	0.57	0.30
Abdominal fat percentage	2.73^a^	1.19^b^	1.58^b^	1.20^b^	0.24	< 0.01

^1^Each value represents the mean of 6 replicates.

The NSP enzymes were provided with 1,500 U xylanase/kg diet and 2,500 U glucanase/kg diet. Protease was provided with 5,000 U/kg of diet.

^a.b^Means with different superscripts within the same column differ significantly (*p* < 0.05).

^2^PC, positive control; NC, negative control; NSP, NSP enzymes addition; SEM, standard error of mean.

### 3.4 Effect of enzyme addition on the digestibility of DM, GE, CP, and AA *in vivo*

Supplementation of the NC diet with NSP enzymes alone or the combination of NSP enzymes plus protease significantly improved DM, GE, and CP digestibility (*p* < 0.01; Table 6). Compared with the unsupplemented NC diet, single-NSP-enzyme supplementation increased DM digestibility by 5.12%, GE digestibility by 2.44%, and CP digestibility by 6.54%, respectively. Furthermore, the combined-enzyme addition increased DM digestibility by 7.28%, GE digestibility by 5.17%, and CP digestibility by 6.45%. The combination of NSP enzyme and protease yielded greater DM and GE digestibility relative to single-NSP-enzyme supplementation (*p* < 0.01). The PC diet exhibited higher digestibility of Met and Lys than the NC diet (*p* < 0.01). Compared with the NC diet, supplementation with NSP enzymes alone increased the digestibility of Thr, Met, Ile, Lys, and Arg by 3.57, 4.87, 4.39, 12.71, and 3.09%, respectively, while the combined-enzyme addition increased their digestibility by 5.69, 7.16, 6.21, 14.15, and 7.23%, respectively (*p* < 0.05).

**Table 6:** Effect of enzyme addition on the digestibility of dry matter (DM), gross energy (GE), crude protein (CP), and amino acids of ducks^1^.

Digestibility (%)^2^	PC	NC	NC + NSP enzyme	NC + NSP enzyme + protease	SEM	*P*-value
DM	84.76^c^	83.74^c^	88.03^b^	89.84^a^	0.52	<0.01
GE	74.62^c^	74.12^c^	75.93^b^	77.95^a^	0.39	<0.01
CP	73.44^ab^	70.83^c^	72.27^ab^	74.18^a^	0.25	<0.01
Thr	71.16^c^	71.24^c^	73.89^ab^	75.80^a^	0.89	<0.01
Met	78.92^ab^	74.07^b^	79.15^ab^	80.94^a^	0.56	0.02
Ile	70.54^c^	73.16^bc^	75.23^ab^	76.69^a^	1.04	0.04
Lys	74.74^a^	69.31^b^	74.82^a^	75.65^a^	0.82	0.01
Arg	72.92^b^	73.18^b^	75.98^ab^	78.80^a^	1.06	0.04

^1^Each value represents the mean of 6 replicates.

The NSP enzymes were provided with 1,500 U xylanase/kg diet and 2,500 U glucanase/kg diet. Protease was provided with 5,000 U/kg of diet.

^a–c^Means with different superscripts within the same column differ significantly (*p* < 0.05).

^2^PC, positive control; NC, negative control; NSP, NSP enzymes addition; SEM, standard error of mean.

## 4 Discussion

Table 7 presents the wide range of nutrient compositions of the energy feedstuffs (wheat, rice, and broken rice) and protein feedstuffs (CSM, RSM, and PNM) from different provinces of China (Table 7). Such variations may be related to differences in the crop varieties, processing methods, growing conditions, and harvesting seasons of cereal grains (16–18). For example, Gutierrez et al. (19) found that drought conditions reduced grain weight, starch, and soluble NSP contents and increased CP content, total arabinoxylans, ADF, lignin, and free sugar contents of wheat. As a result of the dehusking process of rice, broken rice with lower CF, NDF, and ADF contents exhibited greater CP and GE contents than whole rice, as reported previously (20). However, Casas et al. (21) showed that the variations in NDF and ADF contents of broken rice samples were greater than those of cereal grains of whole rice and wheat. Given that different parts and amounts of rice are separated in the milling process, the milling degree may affect the composition of broken rice. For example, under-milled broken rice, which has less germ removed, contains a greater portion of the aleurone layer and germ, resulting in higher nutrient concentrations than well-milled broken rice (22). Similarly, variations in chemical composition were observed in plant protein meal samples (CSM, RSM, and PNM) from different locations and processing conditions. For example, increased autoclaving time in PNM (0, 30, 60, and 90 min) inactivated almost all trypsin inhibitors and significantly reduced weight gain, feed efficiency, and protein solubility in broilers fed corn–PNM diets from 8 to 22 days of age (23, 24). Bifan et al. (25) showed that the free and total gossypol contents, as well as tannin levels in CSM, fluctuated between 0.017 and 0.028%, 0.413 and 0.831%, and 3.13 and 6.75 mg/100 g, respectively, due to differences in geographic origin and processing factors (Table 7). The crude protein content was 2.3–6.1% higher in the “double-zero” RSM than in normal RSM, while the CV of ADF and NDF between double-zero and normal RSM fluctuated from 9.3 to 25.2% (26). Remarkable differences in CP (39.7% vs. 35.6%), CF (7.32% vs. 13.46%), and NDF (24.5% vs. 39.5%) contents were noted between the RSM 1 and RSM 3 samples. We confirmed that the magnitude of variation among sources of ingredients was within the reasonable range observed in previous surveys (21, 22, 27–29). In view of these variations among different feedstuff samples from varying locations and processing conditions, accurate information on their actual nutritional value is required for feed formulation to ensure the quality of complete diets.

**Table 7:** Analyses the composition of wheat, rice, broken rice, cottonseed meal, rapeseed meal, and peanut meal from different locations^1^.

Sample		DM (%)	CP (%)	Ash (%)	EE (%)	CF (%)	Ca (%)	TP (%)	NDF (%)	ADF (%)	GE (MJ/kg)
Wheat^2^	1	90.8	15.6	1.61	1.69	1.84	0.194	0.472	12.30	3.04	16.3
	2	89.9	16.3	1.89	1.49	1.43	0.252	0.657	15.60	3.87	16.2
	3	90.6	15.1	1.80	1.60	2.04	0.343	0.799	15.30	3.56	15.9
	4	91.5	20.0	1.65	1.49	1.66	0.237	0.752	16.14	3.73	15.3
Rice^2^	1	88.2	6.94	4.64	1.62	7.51	0.021	0.411	25.60	14.4	15.2
	2	87.2	7.93	4.83	1.73	9.32	0.032	0.332	28.92	15.6	15.2
	3	87.4	7.53	4.25	1.78	8.95	0.033	0.354	28.04	14.3	14.7
	4	88.0	7.39	4.44	1.84	9.46	0.024	0.362	33.02	16.7	15.4
Broken rice^2^	1	90.3	10.8	1.63	1.73	0.95	0.070	0.371	0.97	0.76	15.2
	2	89.4	10.4	1.87	2.14	1.45	0.062	0.442	1.77	1.00	15.4
	3	89.8	11.5	1.90	1.96	1.55	0.064	0.453	1.79	0.94	15.4
	4	90.1	11.2	1.95	1.94	1.11	0.073	0.434	1.24	0.78	15.2
CSM^2^	1	90.1	42.2	6.03	1.51	14.3	0.210	0.668	38.82	17.7	17.6
	2	90.2	46.8	6.19	1.73	14.7	0.332	0.602	43.24	20.2	17.4
	3	90.8	44.0	6.36	1.68	14.1	0.418	0.841	42.11	18.4	17.4
RSM^2^	1	94.1	39.7	7.38	1.36	7.32	0.572	0.672	24.53	17.8	18.8
	2	89.4	38.3	8.02	1.57	8.02	0.593	0.720	26.82	17.4	18.8
	3	94.5	35.6	8.47	1.30	13.5	0.590	0.680	39.54	17.9	18.9
PNM^2^	1	89.8	46.4	5.32	1.47	6.7	0.351	0.680	20.81	12.4	17.2
	2	94.1	52.8	5.64	1.60	7.5	0.273	0.551	18.92	11.9	17.3
	3	90.4	47.8	5.44	1.86	8.9	0.304	0.611	24.53	13.0	17.9

^1^Wheat samples 1–4 were collected from the Jiangsu, Anhui, Liaoning, and Shanxi provinces of China, respectively; Rice samples 1–4 were collected from Zhejiang, Guangdong, Sichuan, and Jiangxi provinces of China, respectively; Broken rice samples 1–4 were collected from Liaoning, Guangdong, Sichuan, and Jiangxi provinces of China, respectively; CSM samples 1–3 were collected from Sichuan, Hebei, and Jiangsu provinces of China, and the analyzed free gossypol contents were 372, 175 and 989 mg/kg using HPLC method, respectively; RSM samples 1–3 were collected from Jiangsu, Shandong, and Anhui provinces of China, respectively; PNM samples 1–3 were collected from Gansu, Shandong, and Hebei provinces of China, respectively.

^2^The average values represent the measured data in triplicate per sample.

DM, dry matter; CP, crude protein; EE, ether extract; Ca, calcium; TP, total phosphorus; NDF, neutral detergent fiber; ADF, acid detergent fiber; GE, gross energy.

In the present study, supplementation of NSP enzymes improved the IVDMD and IVED of wheat 3, which had the lowest IVED value among the wheat samples, using a two-stage simulated digestion assay for ducks, which was in line with the *in vivo* study reported by Dusel et al. (30) showing that supplementation of enzyme preparations improved the nutritive value of wheat containing low (12.02 MJ/kg) ME levels. This improvement may be attributed to the hydrolysis of encapsulating cell walls, which could improve the accessibility of endogenous enzymes to starch, protein, and fat and enhance energy utilization following NSP enzyme addition. Moreover, NSP enzymes can reduce digesta viscosity and increase nutrient absorption (31, 32). However, no improvements following NSP enzyme addition were observed for the IVDMD and IVED of the screened rice 4, CSM 3, and RSM 3 samples, which could be attributed to differences in the sources, types, and contents of NSPs in plant-derived feedstuffs (33). These inconsistent results also imply that the efficacy of exogenous enzymes exhibits feedstuff specificity. Therefore, it is essential to select proper enzymes for targeted specific substrates to increase feed utilization efficiency. Moreover, the addition of NSP enzymes in combination with protease exhibited greater nutrient digestibility than the addition of NSP enzymes individually in our study, which was consistent with the results reported by Romero et al. (34) demonstrating that a combination of exogenous xylanase, amylase, and protease showed greater improvement in nutrient digestibility compared to supplementation of xylanase plus amylase in broilers. This synergy may occur because the enhanced hydrolysis of structural proteins by protease complements and facilitates NSP hydrolysis (35).

Based on the wheat 3 and RSM 3 samples identified *in vitro*, an *in vivo* experiment was conducted to verify the effects of exogenous enzymes on ther growth performance and nutrient digestibility of ducks fed a corn–wheat–soybean meal–RSM basal diet. The results showed that the addition of NSP enzymes individually or in combination with protease improved the final BW and BWG of Cherry Valley ducks from 14 to 42 days of age, which agrees with previous studies conducted in growing chickens from 4 to 22 days of age (36) and hens from 0 to 126 days of age (37). In addition, exogenous enzyme supplementation decreased the abdominal fat percentage in ducks at 42 days of age. The beneficial effect of exogenous enzyme addition on production performance may be attributed to the increased digestibility of DM, energy, CP, and several AAs in the corn–wheat–soybean meal–RSM basal diet. Moreover, our results demonstrated that the beneficial effect of multiple NSP enzymes plus protease on feed efficiency was superior to that of a single NSP enzyme, which partly agrees with results obtained in broilers (7, 38, 39). This synergistic effect likely occurs because the disruption of the cell wall matrix following the addition of NSP enzymes allows endogenous and exogenous protein hydrolases and greater access to substrates, thereby facilitating release of more AAs for digestion and absorption (35). However, several studies have indicated that there was no beneficial response to exogenous enzyme supplementation on the growth performance and nutrient digestibility of broiler chickens fed diets varying in nutrient density (18, 40). In our study, exogenous enzyme supplementation could not completely restore the decreased growth performance of ducks fed the NC diet to the level of the PC diet with an adequate nutrient level, which was consistent with results in broiler chickens from 1 to 42 days of age (41). This partial response to dietary exogenous enzymes suggests that a greater reduction in the nutritional levels of diets may be necessary to elicit the full compensatory effects of enzyme supplementation (40, 42).

## 5 Conclusion

In conclusion, exogenous enzymes improved the nutrient digestibility of wheat and RSM as well as the corn–wheat–soybean meal–RSM basal diet *in vitro* and *in vivo*, respectively, thereby contributing to the improvement in growth performance of ducks fed a diet with reduced nutrient levels. The consistency of the beneficial effects of exogenous enzymes on nutrient digestibility by exogenous enzymes between *in vitro* and *in vivo* conditions suggest that the *in vitro* assay could be developed into a rapid and reliable method for assessing the efficacy of exogenous enzymes in ducks.

## Data Availability

The original contributions presented in the study are included in the article/supplementary material; further inquiries can be directed to the corresponding authors.
